# Competing Risks Model for Prediction of Small for Gestational Age Neonates and the Role of Second Trimester Soluble Fms-like Tyrosine Kinase-1

**DOI:** 10.3390/jcm10173786

**Published:** 2021-08-24

**Authors:** Urszula Nowacka, Ioannis Papastefanou, Alexandra Bouariu, Argyro Syngelaki, Kypros H. Nicolaides

**Affiliations:** Fetal Medicine Research Institute, King’s College Hospital, London SE5 8BB, UK; ulasarzynska@gmail.com (U.N.); jdpap2@yahoo.co.uk (I.P.); alexandra.bouariu@nhs.net (A.B.); argyro.syngelaki@nhs.net (A.S.)

**Keywords:** second trimester screening, small for gestational age, fetal growth restriction, survival model, Bayes’ theorem, likelihood, soluble fms-like tyrosine kinase-1, pyramid of prenatal care

## Abstract

Small for gestational age (SGA) fetuses/neonates are characterized by the increased risk for adverse outcomes that can be reduced if the condition is identified antenatally. We have recently developed a new approach in SGA prediction that considers SGA a spectrum condition that is reflected in two dimensions: gestational age at delivery and Z score in birth weight for gestational age. The new method has a better predictive ability than the traditionally used risk-scoring systems and logistic regression models. In this prospective study in 40241 singleton pregnancies, at 19–24 weeks’ gestation, we examined the potential value of the antiangiogenic soluble fms-like tyrosine kinase-1 (sFlt-1) and the ratio of sFlt-1 to the angiogenic placental growth factor (PlGF) in the prediction of SGA. We found that first, sFlt-1 did not improve the performance of screening by maternal risk factors, and second, the ratio of sFlt-1/PlGF had a worse performance than PlGF alone in the prediction of SGA. Consequently, second trimester sFlt-1 and sFlt-1/PlGF are not useful in screening for SGA.

## 1. Introduction

Small for gestational age (SGA) fetuses/neonates are characterized by increased risk for stillbirth, morbidities and adverse outcomes that can be substantially reduced if the condition is identified antenatally [1,2,3,4,5,6]. We have recently presented a new approach in SGA prediction that considers SGA a spectrum condition that is reflected in two dimensions: gestational age at delivery (GA_Delivery_) and Z score in birth weight for gestational age (Z_BW_) [7,8,9,10,11,12,13,14]. This new method is a model for the joint distribution of GA_Delivery_ and Z_BW_ that uses the same traditional maternal factors and the already known biomarkers of impaired placentation but in a radically different new way. A continuous *prior* joint distribution of GA_Delivery_ and Z_BW_, according to maternal factors, is combined with a multivariate likelihood of biomarkers according to Bayes’ theorem to obtain a posterior distribution, which allows computation of personalized risks for each patient. A single unified model can be applied at any point of pregnancy, for any desired cut-off in GA_Delivery_ and Z_BW_, enhancing the process of adding a new biomarker. The new method is better than the traditionally used risk-scoring systems and logistic regression models in three aspects: first, predictive ability, second, consistency that has been demonstrated by a process of internal validation, and third, the individualization of risk for each patient and the customization for the local needs of a health care system.

Serum soluble fms-like tyrosine kinase-1 (sFlt-1) is an anti-angiogenic protein involved in the pathophysiology of pre-eclampsia (PE). A large prospective study demonstrated that second trimester sFlt-1 improved only the performance of screening for PE developed before 32 weeks [15]. However, adding sFlt-1 measured at 19–24 weeks improved the prediction of term PE achieved by sFlt-1 at 30–34 weeks [16]. A screening study on 9715 singleton pregnancies has shown that second trimester sFlt-1 is not useful in the prediction of SGA [17].

The objective of this study is to investigate the value of second trimester sFlt-1 in predicting SGA. We modeled sFlt-1 values in relation to both GA_Delivery_ and Z_BW_, jointly and continuously, in the context of the new competing risks model for SGA. We also examined the value of sFlt-1 to placental growth factor (PlGF) ratio at 19 to 24 weeks in the prediction of SGA.

## 2. Methods

### 2.1. Study Population and Design

The data for this study were derived from prospective screening for adverse obstetric outcomes in women attending routine pregnancy care at 19 + 0 to 24 + 6 weeks’ gestation at King’s College Hospital and Medway Maritime Hospital, UK, between 2011 and 2020. In this visit, we first recorded maternal demographic characteristics and medical history. Second, we carried out an ultrasound examination for fetal anatomy and growth. Third, we measured the left and right uterine artery pulsatility index (UtA-PI) either by transvaginal or transabdominal color Doppler ultrasound and calculated the mean value of the two arteries [18,19]. Fourth, we measured the mean arterial pressure (MAP) by validated automated devices and a standardized protocol [20]. The majority of UtA-PI measurements were carried out transvaginally because while we were measuring cervical length, the transabdominal approach was used when women declined transvaginal sonography. The ultrasound scans were carried out by sonographers who had extensive training in ultrasound scanning and had obtained the appropriate Fetal Medicine Foundation Certificate of Competence in ultrasound and Doppler examinations (http://www.fetalmedicine.com, accessed on 1 June 2021). The fetal head circumference, abdominal circumference and femur length were measured, and the estimated fetal weight (EFW) was calculated by the Hadlock formula [21] because a systematic review identified this as being the most accurate model [22]. Gestational age was determined by the measurement of fetal crown-rump length at 11–13 weeks or the fetal head circumference at 19–24 weeks [23,24]. Serum PlGF and sFlt-1 were measured by BRAHMS Kryptor compact PLUS (Thermo Fisher Scientific, Hennigsdorf, Germany), or Cobas e411 (Roche Diagnostics, Penzberg, Germany) between March 2006 and March 2017 at King’s College Hospital and between April 2010 and March 2017 at Medway Maritime Hospital. 

### 2.2. Outcome Measures

Data on pregnancy outcomes were collected from hospital maternity records or the general medical practitioners of the women. The outcome measures of the study were birth of a neonate at or below different thresholds of birth weight percentile for different cut-offs of gestational age at delivery, with or without the occurrence of PE. The obstetric records of all women with pre-existing or pregnancy-associated hypertension were reviewed to determine if the condition was PE, as defined by the American College of Obstetricians and Gynecologists (ACOG) [25]. According to this definition, diagnosis of PE requires the presence of new-onset hypertension (blood pressure ≥140 mmHg systolic and/or ≥90 mmHg diastolic) at ≥20 weeks’ gestation and either proteinuria (≥300 mg/24 h or protein to creatinine ratio >30 mg/mmol or ≥2+ on dipstick testing) or evidence of renal dysfunction (serum creatinine >97 µmol/L), hepatic dysfunction (transaminases ≥ 65 IU/L) or hematological dysfunction (platelet count < 100,000/µL) [25]. The Fetal Medicine Foundation fetal and neonatal population weight charts were used to convert birth weight and EFW to percentiles and Z scores [26].

### 2.3. Statistical Analyses

The recently developed competing risks approach for the prediction of SGA is based on the personalized joint distribution of Z_BW_ and GA_Delivery_ [7,8,9,10,11,12,13,14]. We combined the prior joint distribution of Z_BW_ and GA_Delivery_ with the likelihoods of the biochemical markers, according to Bayes’ theorem, to obtain a pregnancy-specific joint posterior distribution that allows the calculation of risk for any chosen cut-off for Z_BW_ and GA_Delivery_. 

We converted PlGF and sFlt-1 to multiples of the median (MoM) values, as previously described [8,9,10,11,12,13,14]. We calculated the ratio sFlt-1 MoM to PlGF MoM, and we log_10_ transformed it to approximate a Gaussian distribution. Model fitting was carried out within a Bayesian framework using Markov chain Monte Carlo (MCMC) [27]. The statistical software package R was used for data analyses [28].

## 3. Results

The maternal and pregnancy characteristics of the study population that included 40241 singleton pregnancies are provided in Table 1. In the SGA <10th percentile group, when compared with the non-SGA group, there was a lower median maternal age, weight, height and body mass index. There was also a higher incidence of women of Black and South Asian origin, and those with a history of chronic hypertension, cigarette smoking, family history of PE, nulliparity and parous women with PE and/birth of SGA baby in a previous pregnancy, as well as longer interpregnancy interval and incidence of PE or gestational hypertension in the current pregnancy. 

**Figure 1:**
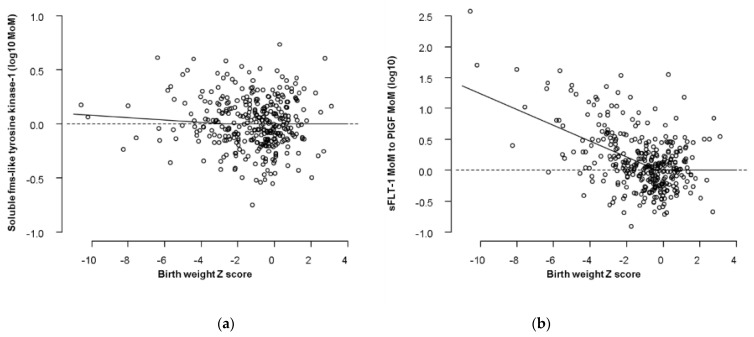
Distribution of biomarkers in relation to Z scores in birth weight. The dots are the cases delivered before 32 weeks and the superimposed regression lines are the ones that corresponds to 28 weeks according to the two-dimensional folded plane models. (**a**) Distribution of sFlt-1; (**b**) Distribution of sFlt-1/PlGF.

**Figure 2:**
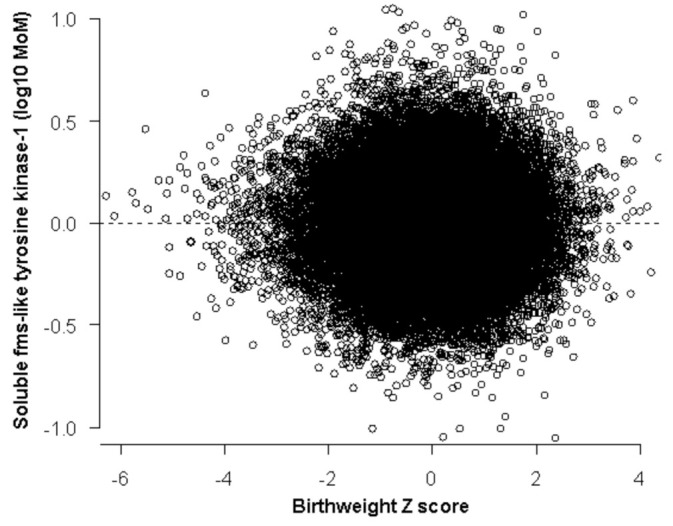
Distribution of sFlt-1 after 32 weeks where no relation to Z scores in birth weight was found.

The distribution of sFlt-1 is described by a folded plain model that revealed a marginal increase for smaller babies born before 32 weeks (Table 2, Figure 1). The sFlt-1 was not related to birth weight after 32 weeks gestation at delivery (Figure 2). Therefore, we did not examine the performance of sFlt-1 after 32 weeks. The sFlt-1/PlGF ratio likelihood had a similar structure to the one that was fitted for PlGF in a previous study [14]. Measurement of sFlt-1 did not improve the prediction of SGA (<10th percentile or <3rd percentile), with or without PE, and delivery at <32 weeks’ gestation provided by maternal factors alone, at a fixed false positive rate of 10% (Table 3). Therefore, we did not examine the combination of sFlt-1 and PlGF with maternal factors. The sFlt-1/PlGF ratio improved the prediction provided by maternal factors, but PlGF combined with maternal factors was better (Table 3). Similarly, sFlt-1/PlGF ratio improved the prediction of SGA born at <37 but not of SGA born ≥37 weeks’ gestation. PlGF alone was consistently better than sFlt-1/PlGF ratio in the prediction of SGA born at <37 or at ≥37 weeks’ gestation (Supplementary Materials Table S1).

## 4. Discussion

### 4.1. Main Findings

There are two main findings of this study that investigated the potential role of second trimester sFlt-1 and the sFlt-1/PlGF ratio in the prediction of SGA. First, sFlt-1 has a marginal trend for higher values for smaller birth weights, but this is confined to babies delivered before 32 weeks’ gestation and does not improve the performance of screening by maternal factors. Second, the ratio of sFlt-1/PlGF has a worse performance than PlGF alone in the prediction of SGA.

This is the first study that examined sFlt-1/PlGF ratio at 19–24 weeks’ gestation in a large sample prospectively collected. We avoided the use of raw values, and we converted biochemical markers to multiples of the median. This method normalizes the skewed distribution of these markers and allows for the use of Bayes’ theorem that requires independence between markers and maternal factors so that their combination is feasible. An important new element in investigating the role of this ratio is the continuous folded plane likelihood in the framework of the new competing risks model for SGA. We have observed that using this ratio compromises the predictive ability of PlGF alone.

### 4.2. Comparison with Previous Studies

In a previous prospective study on 9715 singleton pregnancies, we reported that sFlt-1 measured at 19 to 24 weeks’ gestation was not significantly different in the SGA <5th group born before 37 weeks [17]. In the presented study, we used the new FMF fetal and neonatal weight charts to adjust birth weight for GA_Delivery_. The continuous folded plane likelihood that we developed has shown that there is a trend for increasing sFlt-1 values for lower Z_BW_ until 32 weeks’ gestation. However, this observation was not translated to an improvement in the performance of screening. 

### 4.3. Strengths and Limitations

The strengths of the study are first, the large sample size with prospectively collected data; second, the use of a continuous folded plane likelihood that best describes the distribution of biomarkers and especially that of sFlt-1, which is altered in very small and preterm babies; third, the use of a joint probability model that allows risk computation for any chosen cut-offs; and fourth, the use of Bayes’ rule that allows extension of a single unified model by adding new biomarkers, such as sFlt-1 and sFlt-1/PlGF ratio. We have previously demonstrated that the model is stable and consistent by an internal validation process [7,8,9,12]. Additionally, applying the inferences for the model’s parameters in datasets different than the one that had been used to obtain them has shown that our approach is effective when applied in a new case [14]. We acknowledge the need for external validation of our approach.

## 5. Conclusions

Early identification of SGA aims to reduce the risk of stillbirth and neonatal mortality and morbidity associated with this condition [29]. The use of sFlt-1 measured at 19 to 24 weeks as a biomarker for SGA is restricted by its low deviation in smaller babies and the temporal changes until 32 weeks’ gestation. The use of the sFlt-1/PlGF ratio worsens the performance of screening achieved by PlGF alone. Therefore, second trimester sFlt-1 in either form is unlikely to be clinically useful in the prediction of SGA.

## Figures and Tables

**Table 1:** Maternal and pregnancy characteristics of the study population.

Variables	Total (*n* = 40241)	Non-SGA (*n* = 35468)	SGA (*n* = 4773)	*p*-Value
Maternal age (years)	31.9 (27.9–35.5)	32.0 (28.0–35.5)	31.4 (27.0–35.3)	<0.0001
Maternal weight (kg)	67.2 (59.9–78.1)	68.0 (60.0–79.0)	63.8 (56.4–73.8)	<0.0001
Maternal height (cm)	165 (161–170)	165 (161–170)	163 (158–167)	<0.0001
Body mass index (kg/m^2^)	24.6 (22.0–28.5)	24.7 (22.1–28.6)	24.0 (21.4–27.6)	<0.0001
Gestational age at assessment (w)	21.6 (21.1–22.0)	21.6 (21.1–22.0)	21.6 (21.1–22.0)	0.241
Racial origin				
White	31195 (77.5)	28036 (79.1)	3159 (62.2)	<0.0001
Black	5226 (13.0)	4334 (12.2)	892 (18.7)	<0.0001
South Asian	1923 (4.8)	1487 (4.2)	436 (9.1)	<0.0001
East Asian	784 (2.0)	669 (1.9)	115 (2.4)	0.016
Mixed	1113 (2.8)	942 (2.7)	171 (3.6)	0.0003
Conception				
Natural	38433 (95.5)	33897 (95.6)	4536 (95.0)	0.101
Ovulation induction	295 (0.7)	255 (0.7)	40 (0.8)	0.415
In vitro fertilization	1513 (3.8)	1316 (3.7)	197 (4.1)	0.167
Medical history				
Chronic hypertension	425 (1.1)	323 (0.9)	102 (2.1)	<0.0001
Diabetes mellitus	354 (0.9)	315 (0.9)	39 (0.8)	0.681
SLE/APS	85 (0.2)	68 (0.2)	17 (0.4)	0.031
Cigarette smokers	3016 (7.5)	2324 (6.6)	692 (14.5)	<0.0001
Family history of preeclampsia	1451 (3.6)	1246 (3.5)	205 (4.3)	0.007
Parity				
Nulliparous	18954 (47.1)	16241 (45.8)	2713 (56.8)	<0.0001
Parous with previous SGA	2818 (7.0)	2033 (5.7)	785 (16.5)	<0.0001
Parous with previouspreeclampsia and (or) SGA	3563 (8.9)	2701 (7.6)	862 (18.1)	<0.0001
Inter-pregnancy interval (years)	2.7 (1.7–4.7)	2.7 (1.7–4.6)	3.2 (1.8–5.8)	<0.0001
Preeclampsia	1197 (3.0)	846 (2.4)	351 (7.4)	<0.0001
Gestational hypertension	1095 (2.7)	859 (2.4)	236 (4.9)	<0.0001

Values are given as median (interquartile range) or number (%). Comparisons between outcome groups were performed by chi-square test or Fisher exact test for categorical variables and Mann–Whitney U test for continuous variables. SGA, small for gestational age with birth weight <10th percentile; SLE, Systemic lupus erythematosus; APS = Antiphospholipid Syndrome.

**Table 2:** Fitted folded plane regression model for the mean log_10_ MoM sFlt-1 and mean log_10_ (MoM sFlt-1 / MoM PlGF) conditional to birth weight Z score and gestational age at delivery.

Term	Estimate (Upper and Lower 95 Credibility Limits)	SD
**log_10_ MoM sFlt-1**		
Intercept	−0.028181411 (−0.101200000 to 0.044080000)	0.034100921
Birth weight Z score	−0.011182582 (−0.032760000 to 0.013000250)	0.011519052
(GA–33)^−1^	0.001131449 (−0.001348025 to 0.004564025)	0.001472069
SD for log_10_ MoM sFlt-1	0.233381804 (0.216500000 to 0.252000000)	0.009013250
**log_10_ (MoM sFlt-1/MoM PlGF)**		
Intercept	−0.25555636 (−0.3528000 to −0.1639000)	0.04816489
Birth weight Z score	−0.12802946 (−0.1524000 to −0.1030000)	0.01262894
GA-40	−0.01624357 (−0.0224600 to −0.0102000)	0.00319910
SD for log10 (MoM sFlt-1 / MoM PlGF)	0.31564903 (0.3135000 to 0.3178000)	0.00111485

sFlt-1, serum soluble fms-like tyrosine kinase-1; PlGF, placental growth factor; GA, gestational age at delivery; SD, standard deviation.

**Table 3:** Comparison of detection rate of all SGA (<10th percentile or <3rd percentile), SGA with PE or SGA without PE, with delivery at <32 weeks’ gestation, of different methods of screening at a fixed false positive rate of 10%.

Method of Screening	N	Comparison of Detection by the Two Methods of Screening *n* (%) vs. *n* (%)	Difference in Detection between the Two Methods of Screening *n* (%; 95% CI)	*p*-Value
**<32 weeks**				
**All SGA <10th percentile**				
MF vs MF+ sFlt-1	131	50 (38.2) vs. 51 (38.9)	1 (0.7; −0.7 to 2.1)	0.318
MF vs MF+ sFlt-1/PlGF	131	50 (38.2) vs. 71 (54.2)	21 (16.0; 9.7 to 22.3)	0.0006
MF+PlGF vs MF+ sFlt-1/PlGF	131	81 (61.8) vs. 71 (54.2)	−10 (−7.6; −12.1 to −3.1)	0.016
**SGA <10th percentile with PE**				
MF vs MF+ sFlt-1	43	16 (37.2) vs. 17 (39.5)	1 (2.3; −2.2 to 6.8)	0.347
MF vs MF+ sFlt-1/PlGF	43	16 (37.2) vs. 29 (67.3)	13 (30.1; 16.4 to 43.8)	0.020
MF+PlGF vs MF+ sFlt-1/PlGF	43	30 (69.8) vs. 29 (67.3)	−1 (−2.5; −7.2 to 2.2)	0.057
**SGA <10th percentile no PE**				
MF vs MF+ sFlt-1	88	34 (38.6) vs. 33 (37.5)	−1 (1.2; −3.5 to 1.1)	0.322
MF vs MF+ sFlt-1/PlGF	88	34 (38.6) vs. 48 (54.1)	14 (15.5; 7.9 to 23.1)	0.006
MF+PlGF vs MF+ sFlt-1/PlGF	88	53 (60.2) vs. 48 (54.1)	−5 (−6.1; −11.1 to −1.1)	0.083
**<32 weeks**				
**All SGA <3rd percentile**				
MF vs MF+ sFlt-1	105	41 (39.1) vs. 40 (38.1)	−1 (−1; −2.9 to 0.9)	0.482
MF vs MF+ sFlt-1/PlGF	105	41 (39.1) vs. 60 (57.1)	19 (18; 10.7 to 25.4)	0.0009
MF+PlGF vs MF+ sFlt-1/PlGF	105	71 (67.6) vs. 60 (57.1)	−11 (−10.5; −16.4 to −4.6)	0.021
**SGA <3rd percentile with PE**				
MF vs MF+ sFlt-1	41	16 (39.1) vs. 41 (39.1)	0 (0; −0.2 to 0.2)	1
MF vs MF+ sFlt-1/PlGF	41	16 (39.1) vs. 23 (56.1)	7 (17; 5.5 to 28.5)	0.034
MF+PlGF vs MF+ sFlt-1/PlGF	41	29 (70.7) vs. 23 (56.1)	−6 (−14.6; −25.4 to −3.8)	0.057
**SGA <3rd percentile no PE**				
MF vs MF+ sFlt-1	64	25 (39.1) vs. 24 (37.5)	−1 (−1.6; −4.7 to 1.5)	0.317
MF vs MF+ sFlt-1/PlGF	64	25 (39.1) vs. 37 (57.8)	12 (18.7; 9.2 to 28.3)	0.011
MF+PlGF vs MF+ sFlt-1/PlGF	64	44 (68.8) vs. 37 (57.8)	−7 (−11; −18.7 to −3.3)	0.070

SGA, small for gestational age; PE, preeclampsia; MF, maternal factors; sFlt-1, serum soluble fms-like tyrosine kinase-1; PlGF, placental growth factor.

## Data Availability

Research data are not shared.

## Supplementary Materials

The following are available online at https://www.mdpi.com/article/10.3390/jcm10173786/s1, Table S1: Comparison of detection rate of SGA (<10th percentile or <3rd percentile), with delivery at <37 and ≥37 weeks’ gestation, of different methods of screening at a fixed false positive rate of 10%.
