# Supplementary material for: Competing Risks Model for Prediction of Small for Gestational Age Neonates and the Role of Second Trimester Soluble Fms-like Tyrosine Kinase-1
Source: J Clin Med. 2021 Aug 24;10(17):3786. doi: 10.3390/jcm10173786 (PMC8432206; doi:10.3390/jcm10173786)
Supplement: Supplementary file 1 [file jcm-10-03786-s001.zip › jcm-1307649-supplementary.pdf]

Journal of
Clinical Medicine

MDPI

Table S1. Comparison of detection rate of SGA (<10th percentile or <3rd percentile), with delivery at <37 and ≥37 weeks weeks' gestation, of different methods of screening at a fixed false positive rate of 10%.

Method of screening	N	Comparison of detection by the two methods of screening *n* (%) vs. n (%)	Difference in detection between the two methods of screening *n* (%; 95% CI)	*p*-value
**≥37 weeks**				
**SGA <10th percentile**				
MF vs MF+sFlt-1/PlGF	4014	1268 (31.6) vs. 1212 (30.2)	-56 (-1.4; -1.8 to -1)	<0.0001
MF+PlGF vs MF+sFlt-1/PlGF	4014	1337 (33.3) vs. 1212 (30.2)	-125 (-3.1; -3.6 to -2.6)	<0.0001
**SGA <3rd percentile**				
MF vs MF+sFlt-1/PlGF	1462	547 (37.4) vs. 542 (37.1)	-5 (-0.3; -0.6 to -0.02)	0.4235
MF+PlGF vs MF+sFlt-1/PlGF	1462	594 (40.6) vs. 542 (37.1)	-52 (-3.5; -4.4 to -2.6)	0.00167
**<37 weeks**				
**SGA <10th percentile**				
MF vs MF+sFlt-1/PlGF	759	275 (36.2) vs. 304 (40.1)	29 (3.9; 2.5 to 5.3)	0.017
MF+PlGF vs MF+sFlt-1/PlGF	759	344 (45.3) vs. 304 (40.1)	-40 (-5.2; -6.8 to -3.6)	0.00017
**SGA <3rd percentile**				
MF vs MF+sFlt-1/PlGF	475	176 (37.1) vs. 201 (42.3)	25 (5.2; 3.2 to 7.2)	0.025
MF+PlGF vs MF+sFlt-1/PlGF	475	244 (51.4) vs. 201 (42.3)	-43 (-9.1; -11.7 to -6.5)	<0.0001

SGA, Small for gestational age; MF, maternal factors; sFlt-1 , Serum Soluble fms-like tyrosine kinase-1; PlGF, Placental Growth factor.
